# Supplementary material for: Chromothripsis during telomere crisis is independent of NHEJ, and consistent with a replicative origin
Source: Genome Res. 2019 May;29(5):737–49. doi: 10.1101/gr.240705.118 (PMC6499312; doi:10.1101/gr.240705.118)
Supplement: Supplemental Material [file supp_gr.240705.118_Supplemental_file_1.zip › contigs/annotated_contigs/DB50/contig.3.DB50_length_542_mean_cov_8.46494464945.docx]

**DB50_length_542_mean_cov_8.46494464945**

```
AGAACATACAATGTTTGGTTTTCCATTCCTGAGTTACTTCACTTTGAAAAATAGTCTCCAGTCTCATCCAGGTTGCTGCAAATGCTGTT
 >chr5:164898210-164898391 - E=3e-97 p=0e+00
AATTCATTCCTTTTTATGGCTGAGCAGTATTCCATTGTGTGTATATACATATATCAAAACTGTGATATATCACAGTATTCCATTGTGTG

TAT|CACAGTATTCCATTG|TGGGCATTTGGTATGGTTCCACGA|TTTT|ATAGTTTCAGGTCTTAGCTTTGTGTCCTTAATCCATTTT
                   >chr5:164898076-164898104 - E=3e-05 >chr5:164897074-164897391 - E=
GAGTTGATTTTTGTATAAGGTGAGAGATGAGGATCCAGTTTCATTCTCCTACATGTGGCTAGCCAATTATCCCATTTGTTGAATGGTGT
4e-167
GTCCTTTCCCAACTTTATGGTTTTGTTTGCTTTGTTGAAGATAAGTTGGCTGTAAGTGTTTAGGTTTATTTCTGGGTTCTCTGTTCTGT

TCCATTGGTCTATGTGCCTATTTTTATTTTTATACCAGTACCATGCTGTTTTGGTGAATATGGCCTTATAGTGTAGTTTGAAATCAAGT

AGTGTGATGCCT
```
